# Kampinos National Park: a risk area for spotted fever group rickettsioses, central Poland?

**DOI:** 10.1007/s10493-016-0083-9

**Published:** 2016-09-08

**Authors:** Joanna Stańczak, Beata Biernat, Anna Matyjasek, Maria Racewicz, Marta Zalewska, Daria Lewandowska

**Affiliations:** 1 Department of Tropical Parasitology, Institute of Maritime and Tropical Medicine, Medical University of Gdańsk, Powstania Styczniowego 9B Str., 81-519 Gdynia, Poland; 2 Chair and Clinic of Internal Medicine, Connective Tissue Diseases and Geriatrics, Medical University of Gdańsk, Dębinki 7 Str., 80-211 Gdańsk, Poland; 3 Department of Environmental Hazards Prevention and Allergology, Medical University of Warsaw, Banacha 1a Str., 02-091 Warsaw, Poland

**Keywords:** *Dermacentor reticulatus*, *Ixodes ricinus*, *Rickettsia helvetica*, *Rickettsia raoultii*, Spotted fever group rickettsiae, Seroprevalence, Kampinos National Park, Poland

## Abstract

Ixodid ticks are important vectors of a variety of bacterial and protozoan pathogens which cause infections in humans. In this study, altogether 1041 questing *Ixodes ricinus* (n = 305) and *Dermacentor reticulatus* ticks (n = 736), sympatrically occurring in Kampinos National Park (KPN), central-east Poland, were analyzed by PCR for *Rickettsia* species. Overall, the pathogen prevalence in ticks was 27.5 % for *I. ricinus* and 42.8 % for *D. reticulatus.* Sequencing analysis showed that the first tick species was exclusively infected with *R. helvetica*, whereas the latter was infected with *R. raoultii.* These organism may pose a threat for populations exposed to ticks. Preliminary results of a serosurvey of 74 KPN employees, inhabitants and visitors from the same area showed a 31.1 % total seroprevalence against SFG rickettsiae compared to 13.3 % seropositive blood donors of the control group. Risk factors significantly associated with IgG seropositivity were: occupational exposure to ticks (*p* = 0.002), frequency of tick bites (*p* = 0.02) and male gender (*p* = 0.005). Seropositive and seronegative individuals occupationally exposed to ticks did not differ significantly with respect to age and years of employment.

## Introduction

Spotted fever group (SFG) rickettsioses in humans are caused by small, obligate intracellular Gram-negative bacteria of the genus *Rickettsia* (Rickettsiaceae; Rickettsiales). Most SFG rickettsiae are tick-associated, except *Rickettsia akari* (mite-borne) and *R. felis* (flea-borne). Maintenance of rickettsiae in tick vectors occurs by both vertical and horizontal transmission. Therefore, larvae, nymphs and adults may all be infective for susceptible hosts, including humans. Rickettsiae infecting the ticks’ salivary glands are transmitted to the host during feeding (Brouqui et al. 2007). Thus, ixodid ticks serve both as the main vectors and reservoir hosts for pathogens.

At least eight human rickettsial pathogens circulate in ticks in different and often overlapping parts of Europe, including *R. conorii*, *R. massiliae*, *R. slovaca*, *R. raoultii*, *R. sibirica sibirica*, *R. sibirica mongolotimonae*, *R. helvetica*, *R. rioja*, and possibly others (Eremeeva and Dusch 2015). Four of them (*R. helvetica*, *R. raoultii*, *R. massiliae*, *R. slovaca*) have been so far detected in ticks in Poland (Chmielewski et al. 2009; Mierzejewska et al. 2015; Rymaszewska and Piotrowski 2013; Stańczak et al. 2008). In humans, tick-borne rickettsioses (TBR) have no pathognomonic signs, but may cause a suggesting spectrum of clinical signs: fever, headache, rash, inoculation eschar and enlarged cervical lymph nodes. Acute febrile illness, meningitis and a fatal perimyocarditis, caused by *R. helvetica* have been reported from Sweden (Nilsson 2009; Nilsson et al. 1999, 2010, 2011) and France (Fournier et al. 2000), whereas *R. monacensis* has been isolated so far from three patients with Mediterranean spotted fever-like illness in Spain and in Italy (Jado et al. 2007; Madeddu et al. 2012). *Rickettsia slovaca* and *R. raoultii* are recognized etiologic agent of tick-borne lymphadenopathy (TIBOLA) (Lakos 1997), the disease also known as *Dermacentor*-borne necrosis erythema and lymphadenopathy (DEBONEL) (Ibarra et al. 2006) or scalp eschar and neck lymphadenopathy after tick bite (SENLAT) (Angelakis et al. 2010). Human cases due to *R. slovaca* or, rarely, *R. raoultii* infections have been reported from Hungary, France, Spain, Portugal, Italy and Germany (Lakos 1997; Oteo et al. 2004; Parola et al. 2009; Rieg et al. 2011; Selmi et al. 2008; de Sousa et al. 2013).

According to the Polish regulations the reporting and registration of rickettsioses are obligatory. In 2006-2012, five cases of various SFG rickettsioses, including two imported from South Africa, were reported in Poland. These infections have been recognized in Mazovia (three cases) and Lower Silesia (two cases). Detected rickettsiae have been classified as: *R. conorii*, *R. slovaca*, *R. raoultii* and *R. africae* (Mączka et al. 2013).

The purpose of this work was to evaluate risk of human exposure to *Rickettsia* spp. infection by investigating these bacteria in ticks and antibodies against rickettsiae in individuals presumably exposed to tick bites. For this purpose, a highly frequented recreational area, Kampinos National Park (*Kampinoski Park Narodowy*) (KPN), was chosen as a study area. This is an exceptional national park, as it encompasses forests directly adjacent to Warsaw (Warszawa), the capital of Poland and, as a natural recreational hinterland, is frequently visited by its inhabitants and tourists.

## Materials and methods

### Study area

**Fig. 1:**
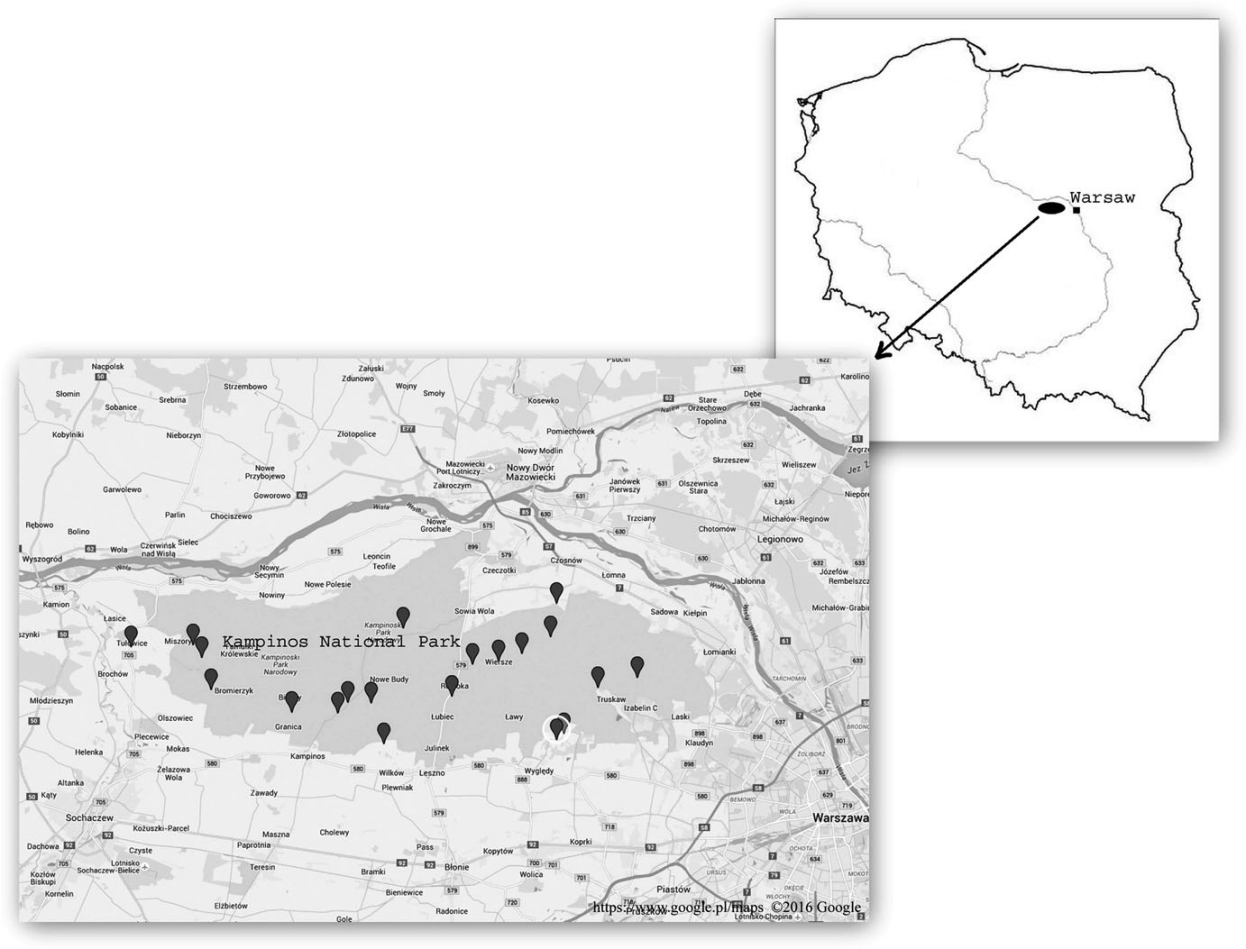
The map of the sampling points in Kampinos National Park, Poland

Kampinos National Park (KNP) [52°18′21″N, 20°36′32″E] (Fig. 1) is the largest natural area in Poland. It covers 38,544 ha, including the ancient Kampinos Primeval Forest (*Puszcza Kampinoska*), and forests account up to almost 73 % of the Park’s surface. In 2000, KPN was added to the list of UNESCO Biosphere Reserves, and it is also a part of the Nature 2000 network. It is characterized by a varied landscape, dominated by two contrasting elements in the direct vicinity—sand dunes and marshes (extensive peat-bogs). The dunes are covered by pine forests, while the peat-bogs by deciduous forests, containing mainly alder cars, and marshy meadows. There are also some areas of wet-ground forest, which add more variety to the forest flora. The park boundaries are open along practically entire length, making extensive human penetration possible. Among visitors walkers represent 27 % of the total. The tourist traffic is of an estimated one million people per year. Moreover, as many as 30 % of “visitors” are illegal pickers of berries and wild fungi. Some area of the Park is owned by farmers, who live in the villages within the park boundaries (together they make ca. 3000 inhabitants). Thus, the contact between people and ticks in the KNP is frequent.

### Tick collection

Questing ticks were collected by the standard flagging method (white flannel blanked 1 × 0.75 m) at 20 different sites (Fig. 1; Table 1) in April–May 2012/2013. In each site, ticks were collected by three persons for 20 min. Attached specimens were removed with forceps and put into sealed vials, each specimen separately. In the laboratory, ticks were killed by rapid immersion in a hot water, identified to species level using standard morphological identification key (Siuda 1993), categorized by site of collection and a developmental stage, and then preserved in 70 % ethanol for further molecular investigations.

**Table 1:** Prevalence of *Rickettsia* spp. in *Ixodes ricinus* and *Dermacentor reticulatus* ticks collected in Kampinos National Park in 2012/2013

Tick species	Developmental stage	No. tested	No. (%) infected
*Ixodes ricinus*	Nymphs	6	0
	Females	155	44 (28.4)
	Males	144	40 (27.8)
	Subtotal	305	84 (27.5)
*Dermacentor reticulatus*	Females	385	163 (42.3)
	Males	351	152 (43.3)
	Subtotal	736	315 (42.8)
Total	Nymphs	6	0
	Females	540	206 (38.1)
	Males	495	193 (40.0)
	Total	1041	399 (38.3)

### DNA extraction

All ticks were analysed individually. Extraction of total DNA was done by boiling crushed specimens in ammonium hydroxide (NH_4_OH) (Guy and Stanek 1991; Rijpkema et al. 1996). Concentrations of DNA were measured with spectrophotometric method (NanoDrop 1000 spectrophotometer, Thermo Scientific, USA). The obtained lysates were stored at −20 °C until use as templates for the molecular investigations.

### Real time PCR

All tick samples were individually screened by real time PCR for the citrate synthase encoding gene (*gltA*) specific for all *Rickettsia* spp. Primers Rick GltA-f (5′-ATCCTACATGCCGATCATGAGC-3′) and Rick GltA-r (5′-GTGAGCAGGTCCCCAAAGTG-3′) were designed to target a 123-bp part of the gene with TaqMan probe (5-HEX-ATGCTTCTACTTCAACAGTCCGAATTGCCG-BHQ1-3′) (Biernat et al. 2016a).

Reaction mixtures and cycling conditions were as previously described (Biernat et al. 2016a). Negative and positive controls were included in all runs. *Rickettsia*-positive control was constructed by cloning the 123-bp PCR amplicon into a circular pJet1.1 plasmids (Fermentas, USA) (Biernat et al. 2016a). Reactions were performed in the Mx3005P Real-Time QPCR System (Stratagene, CA, USA).

### Detection and identification of *Rickettsia* spp.

Most real time PCR-positive samples were subsequently rerun using semi nested PCR and nested PCR assays to obtain longer amplicons for further DNA sequencing. Semi nested PCR was conducted with three primers of which Ric and Ric U8 yielded a 1385-bp fragment encompassing almost the complete 16S rRNA gene, while Ric and Ric Rt flanked a 757 bp fragment (Nilsson et al. 1997). In the nested PCR, primers SLO1F/SLO1R and SLO2F/SLO2R, targeting a fragment (355 bp) of *ompA* gene specific for *R. slovaca* and *R. raoultii*, were used as the outer and inner pairs, respectively (Raoult et al. 2002). Resulted amplicons of 355 bp were considered positive. Additionally, a few of the positive samples were analyzed by the conventional PCR using the primer pair RpCS.877p and RpCS.1258n amplifying a fragment of the citrate synthase encoding gene (*gltA*), which has conserved regions shared by all known *Rickettsia* species (Regnery et al. 1991). DNA products of 380 bp were considered to be positive results.

All amplifications were carried out in the GeneAmp^®^ PCR System 9700 (Applied Biosystems, Foster City, CA, USA) as previously described (Biernat et al. 2016b). PCR products were separated on 2 % agarose gels stained with Midori green DNA Stain (Nippon Genetics Europe) and visualized under UV light using the GelDoc–It, Imagine Systems UV™ Transluminator (Upland, CA, USA). *Rickettsia helvetica* and *R. raoultii* positive samples obtained in our previous investigations (Stańczak 2006; Stańczak et al. 2009) and confirmed by the sequence analysis of the PCR products were used as positive controls. Nuclease-free water was added to each run as a negative control.

### DNA sequencing

Chosen positive amplicons were purified using the Clean-Up purification kit (A&A Biotechnology, Gdynia, Poland), sequenced in both directions with the same primers as used in the semi-nested PCR and nested PCR assays with the ABI Prism^®^ Big Dye™ Terminator v.3.1 Cycle Sequencing Kit and then analyzed with the ABI PRISM 3130 *xL* genetic Analysers (Applied Biosystem) according to the manufacturer’s protocol. Finally, sequences were edited and compared with each other and with corresponding sequences registered in the GenBank database using the NCBI BLAST program (U. S. National Institutes of Health, Bethesda, Maryland) [http://blast.ncbi.nlm.nih.gov/Blast.cgi]. Then consensus sequences were submitted to GenBank.

## Seroreactivity to *Rickettsia* of population exposed to tick-bite

### Study group

A total of 74 persons differently exposed to tick bites were examined. The group comprised 60 workers of KPN (23 females, 37 males; mean age 47.2 years, range 27–65) and 14 members of their families (9 females, 5 males; mean age: 40.1 years, range 9–65) occupationally and recreationally, respectively, exposed to ticks.

As a control group, 30 blood donors (8 females, 22 males; mean age 29.8 years, range 18–60) who denied a tick bite 6 months prior to the investigation were examined. They were city dwellers with no Lyme borreliosis history.

Blood samples were taken by venipuncture and sera separated by centrifugation. In addition, EDTA blood samples were obtained from the study group for real time PCR analysis. Samples were stored at -20 °C until the time of analysis.

### Serological tests

#### Immunoenzymatic assay

The commercial ELISA kits (Spotted Fever Group Rickettsia EIA IgG and IgM Antibody Kit, Fuller Laboratories, Fullerton, CA, USA) were used to detect IgG and IgM antibodies against SFG *Rickettsia* spp. The EIA module wells in this kit utilized a SFG-specific lipopolysaccharide (rLPS) antigen extracted from a members of the SF group, including *R. rickettsii*, *R. akari*, *R. parkeri*, *R. felis*, *R. montanensis*, and others. The tests were carried out according manufacturer’s instructions, including the cutoff calibrator instructions. Absorbance was measured at a wave length of 450 nm on a microtiter plate reader. The obtained values of tested samples were divided by the mean absorbance values of Cutoff Calibrator. The Calibrator was set at an index of 1.0. Index values from 0.8 to 1.2 were considered equivocal (weak positive), above 1.2 as positive and those below 0.8 were considered negative. For analysis, the weak positive results were included into positive group.

#### Micro-immunofluorescence assay (MIF)

To detect IgG antibodies to selected antigens of SGF rickettsiae simultaneously, Rickettsia 2-Antigen MIF IgG Antibody Test (Fuller Laboratories) was used. Purified, acetone-fixed antigens of *R. helvetica* and *R. raoultii* used as an individual substrate on the same slide wells were applied as diagnostics antigens. These slides contain Vero-76 cells with 30–40 infected cells per field when using a 40X lens. The positive and negative controls of human serum used in the procedures were contained in the MIF kits. The assays were performed according to the manufacturer’s instructions. Positive control serum was tested in serial dilution to determine their endpoint titer. Fluorescence of the rickettsiae with an intracellular distribution and intensity pattern similar to the positive control was considered as a positive reaction. The test titer started at 1:32 and an antibody titer of ≥1:64 was considered as positive reaction. All IFA slides were screened by the trained person using a fluorescence microscope (Zeiss).

### PCR assay

EDTA blood samples were screened using real time PCR. DNA extraction was carried out with the Blood Mini kit (A&A Biotechnology, Gdynia, Poland) template preparation according to the manufacturer’s instruction. Obtained templates were stored in −20 until used for real time PCR assays with the same primers and probe like in the case of ticks investigation.

### Seroreactivity and association with risk factors

Each serum donor answered a questionnaire regarding gender, age, ticks exposure (occupational, recreational) and tick bites experienced during the last 12 months (‘none’, ‘1-5’, ‘6-10’, and ‘>10’), length and character of employment in the forest, other tick-borne diseases history, and symptoms such as: an eschar at the site of a tick bite, unexplained fever, headache, myalgia or enlarged lymph nodes 4–6 weeks prior to investigation.

### Ethical statement

The study was approved by the Bioethics Committee of Warsaw Medical University (KB/189/2013). All individuals who agreed to participate signed their consent form and their personal information was held by Department of Tropical Parasitology, Medical University of Gdańsk.

### Statistical analysis

All statistical analyses were performed with R (2008; http://www.R-project.org) and Excel. Qualitative variables were presented using frequencies. Regression analysis and analysis of variance with Tukey’s multiple comparisons of means for quantitative variables and Fisher’s Exact test for qualitative variables were (α = 0.05).

## Results

### Ticks and tick infection rates

Altogether 1041 questing ticks were collected during their spring activity season (April–May) in 2012/2013 at 20 different collection sites spread all-over KPN. Of them, 736 were identified as *D. reticulatus* (70.7 %) (385 females and 351 males) and 305 as *I. ricinus* (29. 3 %) (155 females, 144 males, 6 nymphs) (Table 1). *Dermacentor reticulatus* ticks were found at 16 collection sites whereas *I. ricinus* in 18 of them (Table 2). The first species (93.5 % collected specimens) prevailed in the open areas, meadows, pastures and wastelands (10 collection sites). The second species (62.3 % collected specimens) was dominant in the forested areas (8 collection sites). In two collection sites (mixed stands) both species occurred in comparable numbers (in total 30 vs 25).

**Table 2:** *Rickettsia* spp. prevalence in ticks collected in different sites of Kampinos National Park in 2012/2013

Collection site	Position NE	*I. ricinus*	*D. reticulatus*	Total
		No. tested/no. (%) infected	No. tested/no. (%) infected	No. tested/no. (%) infected
Famułki Brochowskie	52.3102, 20.3637	21/6 (28.6)	1/0	22/6 (27.2)
Górczyńska Droga	52.3022, 20.5045	18/5 (27.7)	0	18/5 (27.7)
Górki—Zamczysko	52.3017, 20.5287	41/10 (24.4)	5/1 (nc)	46/11 (23.9)
Granica	52.2960, 20.4466	17/8 (47.1)	63/19 (30.2)	80/27 (33.8)
Janówek	52.3433; 20.7132	3/1 (nc)	14/6 (42.9)	17/7 (41.2)
Kiścinne	52.3261; 20.6328	1/0 (nc)	104/52 (49.5)	105/52 (49.1)
Adamówek + Łosia W.	52.3642; 20.7192	25/6 (24.0)	133/38 (28.6)	158/44 (27.8)
Korfowe	52.2762; 20.5416	43/8 (18.6)	0	43/8 (18.6)
Łąki Tułowickie	52.3369; 20.2813	0	12/8 (66.7)	12/8 (66.7)
Mariew	52.2826; 20.7272	23/6 (26.1)	27/12 (44.4)	50/18 (36.0)
Miszory	52.3381; 20.3455	11/3 (27.3)	132/73 (55.3)	143/76 (53.1)
OOŚ Narty	52.2953, 20.4940	27/7 (25.9)	0	27/7 (25.9)
Roztoka—Parking	52.3061; 20.6117	13/5 (38.5)	0	13/5 (38.4)
Sieraków	52.3178; 20.8025	12/4 (33.3)	13/7 (53.8)	25/11 (44.0)
Stara Dąbrowa	52.3487; 20.5618	15/6 (40)	1/0	16/6 (37.5)
Truskaw	52.3114; 20.7619	12/2 (16.7)	1/0	13/2 (15.4)
Truskawka	52.3328; 20.6836	1/0	53/25 (47.2)	54/25 (46.3)
Wiersze	52.3282; 20.6601	8/2 (nc)	55/17 (30.9)	63/19 (30.2)
Władysławów	52.3302; 20.3541	0	46/11 (23.9)	46/11 (23.9)
Wólka	52.2788, 20.7192	14/5 (35.7)	76/46 (60.5)	90/51 (56.7)
Total		305/84 (27.5)	736/315 (42.8)	1041/399 (38.3)

*nc* not calculated (no. ticks tested <10)

All collected ticks were individually screened for the presence of *Rickettsia* spp. by the real time PCR and rickettsial DNA was detected in 38.3 % (n = 399) of them. However, the rate of infection differed by tick species. The infection rate of *I. ricinus* was 27.5 % (n = 84/305), being comparable in females and males (28.4 vs 27.8 %), whereas none of 6 nymphs was positive (Table 1). Prevalence of *Rickettsia* spp. in *D. reticulatus* ticks—42.8 % (n = 315/736)—was significantly higher than in *I. ricinus*. The infection rate in male and female ticks also were similar (43.3 vs 42.3 %) (Table 1).

Depending on collection site, the percentage of infected *I. ricinus* varied between 16.7 and 47.1 %, while in the case of *D. reticulatus* the infection level ranged from 18.8 to 66.7 % (Table 2).

### Identification of *Rickettsia* spp.

To identify species of *Rickettsia*, majority of real time PCR-positive samples were subsequently rerun using nested or/and semi nested PCR targeting two genes, 16S rRNA and *ompA*. As a result, among positive *D. reticulatus* 59 amplicons of 16S rRNA-fragment of *Rickettsia* spp. and 223 amplicons of *ompA*-fragment were obtained. Of them, a total of 60 samples were sequenced and 55 obtained sequences were compared with those from rickettsia species and strains deposited in GenBank database. Thirty-three sequences showed 100 % homology with the sequences of partial cds of 16S rRNA gene of 4 *R. raoultii* strains or isolates: strain RpA4 (acc. no. AF120026) (tick, Russia), strain DnS28 (acc. no. AF120024) (tick, Russia), strain Marne (acc. no. DQ365809) (*D. reticulaus*, France) and isolate BL029-2 (acc. no. KJ410261) (*Hyalomma asiaticum*, China). The representative sequence was submitted to GenBank under the accession no. KX024760. Twenty sequences were 100 % identical with numerous homologous fragments of *ompA* gene of *R. raoultii* (former *Rickettsia* sp. RpA4), including *Rickettsia* sp. RpA4 (ac. no. AF120022) detected for the first time in hard ticks in Russia, isolate TG82 (acc. no. KT895942) (*D. marginatus*, Austria), strain Mol 11-06 (acc. no. JX978435) (*Dermacentor* sp., Moldova), clone OrecchiellaOZ77 (acc. no. KC700054) (*D. marginatus*, Italy) and isolate T6 (acc. no. JQ798907) (TIBOLA patient, Hungary). The representative sequence was submitted to GenBank under the accession no. KX051401.Two sequences of *ompA* gene showed homology amounting to 99.8 % with *Rickettsia* sp. RpA4 (ac. no. AF120022) from which differed by one (T → C in position 163) and two nt (A↔G in positions 172 and 360). These sequences were deposited in the GenBank under the acc. nos. KX051403 and KX051402, respectively.

On the other hand, no positive results were obtained in *I. ricinus* ticks analyzed with the nested PCR targeting *ompA* gene. As this gene seems to be not amplified for *R. helvetica* (Roux et al. 1996), negative results suggested that tested *I. ricinus* were infected with this very species. To confirm this suggestion 44 chosen products of 64 semi nested-positive samples were sequenced. The sequence of partial cds 16S rRNA gene obtained from all PCR products matched in 100 % that of *R. helvetica:* clone CsFC (acc. no. GQ413963) (human patient, Sweden), strain IR-671.2-TM (*I. ricinus* feeding on *Turdus merula*, Poland), strain IR-698.9-AF (*I. ricinus* feeding on *Apodemus flavicollis*, Poland). The consensus sequence was submitted to GenBank under the accession no. KX024759.

Additionally, randomly chosen positive samples of *D. reticulatus* (n = 3) and *I. ricinus* (n = 3) were analyzed by the conventional PCR with primers specific for a gene encoding the citrate synthase *gltA* (RpCS.887p and RpCS.1258n6) (Regnery et al. 1991) and resulted amplicons of ~380 bp were applied for sequencing. Three sequences derived from *D. reticulatus* had 100 % similarity to the *gltA* gene of *R. raoultii* isolate S2 (acc. no. LC060713) (*D. reticulatus*, Hungary, Germany), strain T3 (acc. no. KT895941) (*D. reticulatus*, Austria) and strain Alashankou-112 (acc. no. KT261764) (*D. marginatus*, China). They differed by one nucleotide (C → A) from *R. raoultii* strain Marne (acc. no. DQ365803) (*D. reticulatus*, France) and strain Khabarovsk (acc. no. DQ365804) (*D. silvarum*, Russia). The representative sequence was deposited in Genbank under the accession no KX051404.

Sequences of the *gltA* gene fragment from the three *I. ricinus* samples (GenBank acc. no. KX051405) were identical to *R. helvetica* 6DI76 isolate and 99Bc strain sequences (acc. no. KC007126; JX0406636) from *I. ricinus* from Germany and Romania.

### Seroprevalence

All 104 sera of the study (n = 74) and control (n = 30) groups were tested for the presence of the IgM and IgG antibodies against SFG rickettsiae. Of them, the IgM antibodies were detected only in one person of the study group (1/74; 1.4 %), a woman recreationally exposed to tick bites, who denied a tick bite at least 2 month prior investigations and did not complain of any symptom characteristic for a rickettsiosis. Moreover, none of the other examined participants reported flulike symptoms, showed fever and/or rash typical for the clinical form of SFG rickettsioses.

Among 74 persons of the two study groups, employees of KPN and the recreational group, the presence of IgG antibodies was found in 35 and 14.3 % respectively. This difference, however, was statistically insignificant (*p* = 0.2). In the control group of blood donors, the frequency of positive results was of 13.3 %, significantly smaller than in the occupationally exposed group (*p* = 0.04) and similar to the recreational group (Table 3).

**Table 3:** Prevalence of IgG against SFG rickettsiae antigen in the KPN employees differently exposed to tick bites, recreational and control groups according to gender

Study groups	No. tested/no. (%) seropositive		
	Females	Males	Total
KNP employees with outdoor activity	5/2 (40)	23/12 (52.2)	28/14 (50.0)
KNP employees with indoor activity	18/0 (0.0)	14/7 (50.0)	32/7 (21.9)
Subtotal	23/2 (8.7)	37/19 (51.4)	60/21 (35.0)
Recreational group	9/2 (22.2)	5/0 (0.0)	14/2 (14.3)
Total	32/4 (12.5)	42/19 (45.2)	74/23 (31.1)
Control group^a^	8/1 (12.0)	22/3 (13.6)	30/4 (13.3)

^a^Blood donors

Separately, the prevalence of antibodies within the group of the KNP employees with outdoor activity (foresters, forestry rangers, forestry workers, etc.) (50 %) was 2.3 times greater than the positivity rate in the group of office workers (*p* = 0.03) (21.9 %) who only occasionally visit forests doing duty and 3.5 times higher than in other individuals exposed to ticks during leisure activity (14.3 %); the difference was statistically significant (*p* = 0.04). Also differences observed in percentage of positive results in male (51.4 %) and female (8.7 %) KPN participants were statistically significant (*p* = 0.002) (Table 3).

Within all study groups, a total of 57 (77 %) individuals declared at least one tick bite in the year before the study; majority (n = 54) up to 5 bites. The total positivity rate observed among them (36.8 %) differed significantly (*p* = 0.008) from the positivity rate (11.8–13.3 %) noted in the group of people (n = 47) who denied the tick bite, including 30 persons of the control group. Among employees of KPN, those who denied tick bites in majority consisted of office workers (n = 15/16; 93.7 %). On the other hand, percentages of seropositive KPN workers did not differ significantly with respect to age (*p* = 0.6) (range 26–70 years) and total years of employment (*p* = 0.6) (range 0.5–45 years). However, it should be taken into consideration that the groups categorized according to different variables were too small for definitive statements.

### Microimmunofluorescence

MIF test was employed to confirm the presence of anti-*R. helvetica* and/or anti-*R. raoultii* IgG antibodies in ELISA-positive sera and IgG titer ≥64 were considered positive. In serial dilutions from 1:32 to 1:512, the highest detected titer reached 128. This result concerned the only person who tested positive IgM in ELISA test. In 28/34 sera, strong specific fluorescence was observed in titer 1:64 which corresponded with positive ELISA IgG results. Weaker fluorescence in titer 1:64 was observed in 4 sera whereas lower level of antibodies (titer 1:32) was found in one serum sample. All these results corresponded with equivocal ELISA IgG results.

Observed cross-reactivity made differentiation between *R. helvetica* and *R. raoultii* unable.

### PCR assay

None of the blood samples collected from participants was found to be PCR positive for *Rickettsia* spp.

## Discussion

*Ixodes ricinus* is the predominating tick species in Poland, distributed throughout the country. It occurs mainly in deciduous and mixed forests, or bushy thickets. *Dermacentor reticulatus* is found mainly on the eastern side of the Wisła (Vistula) river: in the north-eastern and eastern parts of Poland. Recent studies, however, have shown that its range of occurrence is much more extended in western Poland than expected. The north–south strip (belt) in the center of the country seems to be free of this species and is known as “the gap” which divides ticks into two separated populations—western and eastern. In our country *D. reticulatus* exists mainly in open habitats such as mixed swamp forests, mid-forest glades and meadows, clearings and bushy pastures on small hills among marshes covered with gray willow (Nowak-Chmura and Siuda 2012).

Diverse landscape, reach and variable vegetation, as well as a variety of host animals, including the elk (*Alces alces*), a symbol of KPN, create in the Park favorable environmental conditions for the development and survival both of *I. ricinus* and *D. reticulatus.* Results of the present studies confirmed a sympatric occurrence of these two species on the whole area of KNP and the extending range of the meadow ticks on the western side of the Wisła river. Moreover, they showed a high overall prevalence, exceeding 38 %, of SFG *Rickettsia* spp. infection in ticks collected from vegetation. The mean infection rate in *D. reticulatus* (~43 %) was in agreement with the prevalence range of the pathogen (~40–53 %) previously observed in eastern and western population of *D. reticulatus* in Poland (Mierzejewska et al. 2015; Stańczak 2006; Wójcik-Fatla et al. 2013) and reported from neighboring Belarus (44.5 %) (Reye et al. 2013) and Germany (56.7 %) (Silaghi et al. 2011). On the other hand, it was much higher than rickettsial infection determined in *D. reticulatus* in Wales and England (27 %) (Tijsse-Klasen et al. 2011), Slovakia (22.3–27 %) (Špitalská et al. 2012), Hungary (26.8 %) (Sréter-Lancz et al. 2006), and in the Netherlands (6 %) (Hofmeester et al. 2015).

In questing adult *I. ricinus*, the observed prevalence of *Rickettsia* spp. of ~28 % was higher than that previously reported in *I. ricinus* ticks from other areas of Poland (1–11.1 %) (Stańczak et al. 2008; Welc-Falęciak et al. 2014), Austria (16.8 %) (Sonnleitner et al. 2013), Germany (11.7–13.7 %) (Silaghi et al. 2011), Slovakia (6.1–11.7 %) (Špitalská et al. 2012, 2016; Švehlová et al. 2014), Wales and England (6.5 %) (Tijsse-Klasen et al. 2011), Sweden (1.5–17.3 %) (Severinsson et al. 2010) and Finland (1.5 %) (Sormunen et al. 2016). It is worth mentioning, however, that an exceptionally high infection rate of *I. ricinus* with rickettsiae (52.5 %) was reported in the city of Hamburg, Germany (May and Strube 2014) and in a vegetation-rich dune area in The Netherland: ~66 % (Sprong et al. 2009). All these reports reflect a great spatial variation in prevalence of *Rickettsia* spp. in European tick populations.

Sequence analysis of fragments of 16S rRNA, *ompA* and *gltA* genes allowed the identification of *Rickettsia* species. *D. reticulatus* was found to be almost exclusively infected with *R. raoultii* (99.8-100 % homology) whereas *I. ricinus* with *R. helvetica* (100 % identity). These findings are in accordance with results from other European studies, including Slovakian (Švehlová et al. 2014), Austrian (Sonnleitner et al. 2013) and Swedish (Severinsson et al. 2010) investigations.

*Dermacentor reticulatus* was proved to be the competent vector of *R. raoultii* with a high level of transovarial (90 %) and transstadial transmission (98 %) (Samoylenko et al. 2009). Although *R. raoultii* seems to be the predominant *Rickettsia* species in meadow ticks, they also may harbor *R. slovaca* and *R. helvetica* (Dobec et al. 2009; Dobler and Wölfel 2009; Tijsse-Klasen et al. 2013; Špitalská et al. 2012). On the other hand *I. ricinus* is considered the major reservoir host (Sprong et al. 2009) and vector for *R. helvetica*, with the transovarial transmission rate up to 100 % (Socolovschi et al., 2009). In Poland, however, and nearby countries this tick is found to be infected also with other *Rickettsia* species: *R. monacensis* (Dobler et al. 2009; Reye et al. 2013; Rymaszewska and Piotrowski 2013; Welc-Falęciak et al. 2014; Simser et al. 2002; Sormunen et al. 2016) and, rarely, with *R. rauoltii* and *R. slovaca* (Chmielewski et al. 2009). Moreover, the occurrence of *R. massiliae*, *R. felis* and *Rickettsia* sp. similar to *R. bellii* was reported in *I. ricinus* in Germany (Dobler and Wölfel 2009; Sprong et al. 2009).

Evidence of the presence of *R. raoultii* and *R. helvetica* in ticks and observed high infection level indicate potential epidemiological and epizootiological significance of *D. reticulatus* and *I. ricinus* in Kampinos National Park. To answer the question whether autochthonous transmission of rickettsiae to humans may occur in this area, we conducted a preliminary study of the presence of antibodies against *Rickettsia* spp. in groups of people differently exposed to ticks. We have shown that seropositivity to rickettsiae was common among them. The prevalences ranged from 13.3 to 50 % were recorded among KNP employees with outdoor activity and in office workers who occasionally visit forests doing duty, in individuals exposed to ticks during leisure activity and in the group of blood donors. This suggests that contacts between ticks and humans, and transmission of *Rickettsia* spp. is frequent. Moreover, persons who denied tick bites at least 6 month prior to the investigation had a seropositivity rate for IgG of 11.8–13.3 %, that confirms once more the well-known clinical observation that tick bites often go unnoticed.

The overall seropositivity rate (31.1 %) detected in the present study is comparable with that reported in studies performed in eastern Poland, were total 36 % of forestry and agricultural workers were found to be positive (Zając et al. 2013). As arthropod bites and arthropod-borne infections are the frequent occupational hazards among forestry workers, in both studies the seroprevalence was found to be the highest among forestry workers with outdoor activity—50 and 50.7 %, respectively. These results were significantly higher than the result obtained for forestry workers from northeastern and southern Poland (14.7 %) (Podsiadły et al. 2011) as well as from north-eastern Italy (3.9 %) and Alsace in France (9.2 %) (Cinco et al. 2006; Fournier et al. 2000). In Germany, antibodies against different *Rickettsia* spp. were found in 9.1 % hunters (Jansen et al. 2008). Survey of another risk group, military recruits during their field training period in the highly tick endemic area of Gotland in Sweden, showed that 22.9 % of them had antibodies against *R. helvetica* (Nilsson et al. 2005). The latter result is comparable with the seroprevalence of KPN employees with the advantage of indoor activity.

Moreover, a serosurvey conducted in Danish patients seropositive for Lyme borreliosis showed that 12.5 % of them had positive antibody titers to *R. helvetica* (Nielsen et al. 2004). Finally, in southern Sweden, 10 % patients with erythema migrans (EM) and/or general signs of infection following a tick bite had antibodies against the same rickettsial species (Lindblom et al. 2013). These results correspond with the seropositivity level among KPN visitors, recreationally exposed to the tick bites.

On the other hand, SFG *Rickettsia* antibody prevalence in blood donors varies as well. In Sweden approximately 1 % of them were seroreactive (Lindblom et al. 2013), whereas in Tyrol, Austria, seroprevalence ranged from 4.8 to 10.6 % (Sonnleitner et al. 2013), and in our control group reached 13.3 %.

To explain so high seroprevalence in people occupationally exposed to ticks in eastern Poland, 36 % of the total, Zaja˛c et al. (2013) suggested that they lived in the area where over 50 % of *D. reticulatus* ticks harbored *R. raoultii* (Wójcik-Fatla et al. 2013) and thus were under significantly increased risk of infection with these rickettsiae. The only case of autochthonous spotted fever described in Poland was cause the most probably by *R. raoultii* (Świtaj et al. 2012), what may support this suggestion. Also in KNP prevalence of *D. reticulatus* infected with *R. raoultii* exceeded 40 %. However, *D. reticulatus* rarely bites humans. None of the individuals surveyed in our study declared to be bitten by this species, although some of them occasionally found these ticks crawling on their clothes. In contrary, majority of them reported *I. ricinus* bites with a frequency of 1–5 per year. This tick species shows a high affinity for humans and in Poland, of SFG rickettsiae, is almost exclusively infected with *R. helvetica* (Stańczak et al. 2008). Interestingly, prevalence of *R. helvetica* in *I. ricinus* in KPN (27.5 %) was comparable or lower than seroprevalence rates in humans differently exposed to tick bites (range 14.3–50 %). This may suggest that seroprevalence in humans does not directly reflect the prevalence in ticks.

In conclusion, on multivariate regression analysis risk factors significantly associated with SFG rickettsiae infection (prevalence of antibodies) were: occupational exposure to ticks (*p* = 0.02), male gender (*p* = 0.004) and frequency tick bites (*p* = 0.02).

Unfortunately, results of the *R. helvetica*- and *R. raoultii*-specific MIF test did not answer the question which of these two rickettsiae evoke positive immune response in studied groups. MIF has been the serologic gold standard, originally being the means of determining separate species, however **S**FG rickettsiae cause cross-reactions within the group (Fournier et al. 2000; Hechemy et al. 1989). We did not observe a significant difference in specific fluorescence between *R. helvetica* and *R. raoultii*, and uncertainty about the source of the infection remained. Interestingly, Podsiadły et al. (2011) detected by MIF antibodies to *R. massiliae* in 79 % of the seropositive forestry workers from northeastern and southern Poland. However, *R. massiliae* is commonly found in *Rhipicephalus sanguineus* or *R. turanicus* ticks (Cardeñosa et al. 2003) which are absent in Poland. Recently it has been detected in *Haemaphysalis punctata* (Tijsse-Klasen et al. 2013), which may attack humans and is recognized as existing permanently in Poland, but in West Pomerania province (Nowak-Chmura and Siuda 2012).

The lack of IGM and the presence of IgG antibodies against SFG *Rickettsia* in studied groups in titer 1:64 reflect infection acquired at an undetermined time. The past infection was also confirmed by negative results of the real time PCR assay. Actually, a rickettsemia has been demonstrated to occur on the first stage of the disease. None of the forestry workers and other individuals in this study reported any tick bite-related symptoms at least 6 weeks prior to the investigation, however, subclinical infection should not be excluded.

These findings confirm that rickettsial tick-transmitted agents are widely distributed in Poland and suggest that they should be taken into consideration in the differential diagnosis of febrile patients with a recent history of tick bite in the investigated area and other regions of Poland. The prevalence of rickettsial diseases in Poland is probably underestimated. To prove, however, that spotted fever rickettsioses occur in the country, the isolation of the agents from patients is needed. The results also demonstrate a need for further, more extensive studies.
